# Nickel (II) Preconcentration and Speciation Analysis During Transport from Aqueous Solutions Using a Hollow-fiber Permeation Liquid Membrane (HFPLM) Device

**DOI:** 10.3390/membranes1030217

**Published:** 2011-08-18

**Authors:** Ana Nelly Bautista-Flores, Eduardo Rodríguez de San Miguel, Josefina de Gyves, Jan Åke Jönsson

**Affiliations:** 1 Química Industrial, Universidad Tecnológica del Sureste de Veracruz, Av. Universidad Tecnológica Lote Grande #1, Nanchital 93770, Veracruz, Mexico; E-Mail: bafa27@hotmail.com; 2 Departamento de Química Analítica, Facultad de Química, UNAM, Ciudad Universitaria, México 04510, D.F., Mexico; E-Mail: degyves@unam.mx; 3 Center for Analysis and Synthesis, Department of Chemistry, Lund University, P.O. Box 124, Lund, SE-221 00, Sweden; E-Mail: Jan_Ake.Jonsson@organic.lu.se

**Keywords:** nickel, preconcentration, speciation analysis, bioavailability, permeation liquid membrane, hollow fiber

## Abstract

Nickel (II) preconcentration and speciation analysis using a hollow fiber supported liquid membrane (HFSLM) device was studied. A counterflow of protons coupled to complexation with formate provided the driving force of the process, while Kelex 100 was employed as carrier. The influence of variables related to module configuration (acceptor pH and carrier concentration) and to the sample properties (donor pH) on the preconcentration factor, E, was simultaneously studied and optimized using a 3 factor Doehlert matrix response surface methodology. The effect of metal concentration was studied as well. Preconcentration factors as high as 4240 were observed depending on the values of the different variables. The effects of the presence of inorganic anions (NO_2_^−^, SO_4_^2−^, Cl^−^, NO_3_^−^, CO_3_^2−^, CN^−^) and dissolved organic matter (DOM) in the form of humic acids were additionally considered in order to carry out a speciation analysis study. Nickel preconcentration was observed to be independent of both effects, except when cyanide was present in the donor phase. A characterization of the transport regime was performed through the analysis of the dependence of E on the temperature. E increases with the increase in temperature according to the equation E(K) = −8617.3 + 30.5T with an activation energy of 56.7 kJ mol^−1^ suggesting a kinetic-controlled regime. Sample depletion ranged from 12 to 1.2% depending on the volume of the donor phase (100 to 1000 mL, respectively).

## 1. Introduction

Nickel is an essential metal for plants and some animals, and it is widely distributed in the environment. This element enters aquatic ecosystems by dissolution of rocks and soils, atmospheric precipitation and biological cycles. Also, the high consumption of nickel products in industrial activities (electroplating, stainless steel manufacture, and nickel–cadmium batteries) and the disposal of residual waters lead inevitably to environmental pollution with this metal ion. Human exposure to nickel occurs primarily via inhalation and ingestion, being common among metallurgy workers. The accumulation of nickel in the body through chronic exposure can lead to lung fibrosis, cardiovascular and kidney diseases; the most serious concern is related to carcinogenic activity [1].

Natural waters (pristine streams, rivers and lakes) contain 0.2–10 μg L^−1^ total dissolved nickel and surface water near mines and smelters contains up to 6.4 mg L^−1^. Coastal, bay and estuary waters contain nickel concentrations from about 0.20 to 5.3 μg L^−1^ and ocean surface water contains 0.15–0.93 μg L^−1^ [2]. However, these concentrations tend to increase due to contamination; for example, the concentration of nickel in the Mersey estuary in Ukraine has been reported between 0.59 and 13.5 μg L^−1^ [3]. In spite of the determination of total metal concentrations being a common practice, nowadays it is accepted that the bioavailability and toxicity of metal ions cannot be predicted from this parameter alone, but rather from the concentration of several chemical species and the free ion [4]. Therefore, speciation studies of metal ions are highly important to understand their ecotoxicological impact. Concerning speciation studies, it is important to distinguish two different concepts related to the speciation of the elements: speciation and speciation analysis. Speciation refers to the distribution of an element among defined chemical species while speciation analysis refers to analytical activities oriented to identify and/or measuring species [5].

Several techniques have been developed for speciation studies of nickel in aqueous solutions; among them competing ligand-exchange methods [6,7,8], ion exchange [9], diffusion gradients in thin films [8,10,11], capillary electrophoresis [12], and membrane-based techniques (ultrafiltration [11], Donnan membrane technique [8,13], and liquid membranes [8,14,15]). The latter techniques offer excellent performance using simple equipment and the reliability of their results for some metal ions and organic pollutants has been favorably compared with bioavailability tests using model microorganisms [16,17].

Supported liquid membranes (SLM), also named permeation liquid membranes (PLM) to emphasize the transport process rather than their implementation in a defined set-up, were developed within the hydrometallurgical industry and can also be used for the simultaneous separation and enrichment of metals in analytical methods. Their use as preconcentration devices for metal ions has been well documented [18]. In this context, Aouarram *et al.* [19] have reported a bulk SLM system that, under optimal conditions, showed an average preconcentration yield for real seawater samples of 98 ± 5%, with a nickel preconcentration factor of 20.83 and metal concentrations ranging between 2.8 and 5.4 μg L^−1^ using a synthesized carrier. Moreover, Domínguez-Lledó *et al.* [2] have reported a bulk SLM system using pyridine-2-acetaldehyde benzoylhydrazone as carrier for nickel. The preconcentration was achieved through pH control of source and receiving solutions via a counterflow of protons. High transport efficiencies (101.2 ± 1.8%–99.7 ± 4.2%) were obtained in a receiving phase of 0.31 mol L^−1^ nitric acid after 9–13 h depending on sample salinity. Furthermore, special characteristics of SLM systems, e.g., tunable selectivity by incorporating different complexing ligands in the membrane, lability control by changing the hydrodynamic conditions, the possibility of coupling the SLM devices to different types of detectors for *in-situ* monitoring, and their high tolerance to fouling in high dissolved organic matter (DOM) matrices [20,21,22,23], make them suitable for the study of trace metal speciation in natural waters as well.

Bayen *et al.* selected a mixture containing 1,10-didecyl-1,10-diaza-18-crown-6 ether (22DD) and di(2-ethylhexyl) phosphoric acid (D2EHPA) in toluene/phenylhexane as the optimized organic phase in order to determine free Ni^2+^ in the presence of Ni complexes in a flat PLM [14]. This PLM system was shown to be a reliable tool to measure free nickel concentrations down to 10^−7^ M. In another work [24] a mixture of 1,10-dibenzyl-1,10-diaza-18-crown-6 and bis(2-ethylhexyl) phosphate dissolved in hexylbenzene was used for passive sampling of ppb levels of Pb(II), Cu(II), Zn(II), Mn(II), Ni(II), and Cd(II) in water. The target ions were simultaneously transported and preconcentrated into a citric acid acceptor solution within the lumen of a hollow fiber with enrichment factors of 5 to 4000. Nickel showed the lowest enrichment factors. To the best of our knowledge, only the latter study has been reported up to now for the preconcentration and speciation analysis of nickel through an SLM in a hollow fiber configuration in spite of the excellent performance characteristics of these devices.

Consequently, in the present work Ni(II) transport through a hollow fiber permeation liquid membrane (HFPLM) device is studied employing the carrier Kelex 100 (7-(4-ethyl-1-methylocty)-8-hydroxyquinoline). This extractant was selected due to its excellent extraction performance towards Ni in solid-phase extraction cartridges [25], together with its ability to exchange protons, so that the driving force for permeation could be related to the pH gradient between the acceptor and donor phases. Proton-driven systems have shown advantages over sodium-driven ones in Estuarine Water samples with high sodium concentrations [20]; however, few reports concerning the feasibility of use and performance of such systems exist in the actual literature. The influence of different variables that affect the transport properties of the sampling module including some environmental factors are evaluated to contribute in the understanding of the methodological approach for speciation analysis with HFPLM devices.

## 2. Results and Discussion

### 2.1. Pertraction Time

Using preliminary established conditions (donor phase: 6.6 × 10^−6^ mol L^−1^ Ni(II) at pH 7 adjusted with 0.01 mol L^−1^ MES; acceptor phase: 0.01 mol L^−1^ HCOOH at pH 1; membrane phase: 0.03 mol L^−1^ Kelex 100 in n-hexyl-benzene; orbital shaking) the time-depending profile of the preconcentration factor, E, defined as E = [Ni(II)]_acceptor_/[Ni(II)]_donor_, was measured. It was observed that after 240 min a constant value was obtained and further experiments were performed using 300 min of pertraction time.

### 2.2. Optimization of Module Intrinsic Parameters

As the HFSLM device is expected to operate in conditions where the donor solution phase is not under experimental control, e.g., sampling of natural environments as rivers and lakes, the intrinsic variables of the module that can actually be beforehand conditioned are the membrane phase and acceptor solution compositions. However, it is important that under the selected values of these variables the device works properly for different donor pH values. Thus, to simultaneously evaluate the effect of both the experimentally-controlled (carrier concentration and acceptor pH) and the environmentally-imposed (donor pH) variables, an analysis and optimization procedure, using a three-variable Doehlert matrix response surface methodology, were carried out [26]. Response surface methodology is a collection of mathematical and statistical techniques based on the fit of a polynomial equation to the experimental data, which must describe the behavior of a data set with the objective of making statistical previsions. It can be well applied when a response or a set of responses of interest are influenced by several variables. The objective is to simultaneously optimize the levels of these variables to attain the best system performance [27]. Doehlert is a second-order experimental design which defines a specific set of combinations for the levels of variables (design matrix) that must be applied experimentally to obtain the response. For studying the effect of three variables, a plausible experimental matrix including 5, 7, and 3 levels for the variables is shown in Table 1 using coded values. Coding is a simple linear transformation of the original measurement scale that enables simplification in data treatment and evaluation. The relationship between coded and real values of the variables is given by:

(1) $$C_{i}=\left\{\frac{X_{i}-X_{i}^{0}}{\Delta X_{i}}\right\}\theta$$

where C_i_ is the coded value for the level of factor *i*, *X_i_* is its real value in an experiment, $X_{i}^{0}$ is the real value at the center of the experimental domain, *ΔX_i_* is the step of variation of the real value, and *θ* is the coded value limit for each factor [26]. The acceptor pH was varied in the 1.0–5.0 interval (column A, coded values from −1 to 1), Kelex 100 concentrations were varied in the range 12–47 mmol L^−1^ (column B, coded values from −0.866 to 0.866), and the donor phase was varied in the 5.6–7.4 interval (column C, coded values from −0.817 to 0.817).

**Figure 1:**
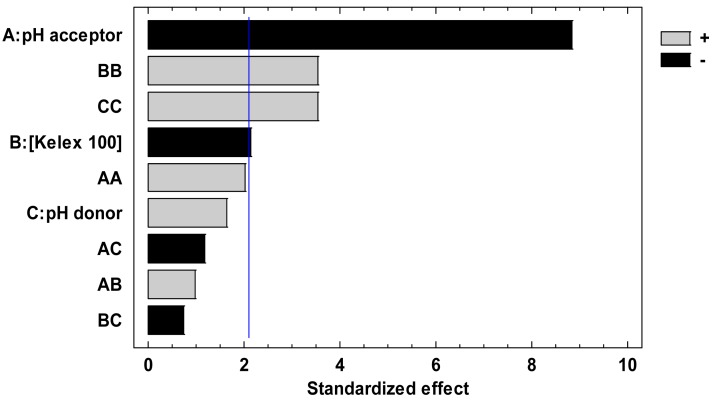
Pareto chart showing the main effects (A: acceptor pH) and interactions of the variables (BB: Kelex 100 concentration, and CC: donor pH) that significantly contribute to E, according to the results of the experimental design matrix shown in Table 1.

After performing the 13 experiments of the Doehlert matrix in the specified coded levels of the three variables in duplicate, and measuring E as response, the pareto chart (Figure 1) and ANOVA analysis (not shown) indicated that all variables significantly contributed to E. According to the figure, at least three effects (A, BB, and CC) are higher than the experimental error denoted by the vertical line indicating its 95% confidence interval.

**Table 1:** Doehlert matrix employed for studying the simultaneous effect of the acceptor pH, donor pH, and Kelex 100 concentration on Ni(II) enrichment factor, E.

**Experiment**	**(A) acceptor pH**	**(B) [Kelex 100]**	**(C) donor pH**
1	0	0	0
2	1	0	0
3	0.5	0.866	0
4	0.5	0.289	0.817
5	−1	0	0
6	−0.5	−0.866	0
7	−0.5	−0.289	−0.817
8	0.5	−0.866	0
9	0.5	−0.289	−0.817
10	−0.5	0.866	0
11	0	0.577	−0.817
12	−0.5	0.289	0.817
13	0	−0.577	0.817

In Figure 1, A stands for the effect of the acceptor pH, while BB and CC represent interaction terms that give rise to Kelex 100 concentration and donor pH quadratic terms in the fitted coded modeling equation:

(2) $$E=138.9-246.6pH_{\text{acceptor}}+164.4{\left[\text{Kelex}100\right]}^{2}+141.3pH_{\text{donor}}^{2}$$

The pH of the acceptor solution contributes as a main effect (linear term) and its increment tends to decrease the E value, according to what is expected, taking into account the reduction in the transport driving force. As for Kelex 100 concentration and the pH of the donor solution, these variables strongly interact within themselves (quadratic terms), as previously discussed, and their effects cause an increase in E. Numerical analysis performed to find optimal conditions for maximizing E resulted in the coded values of −1 (pH = 1) for the acceptor pH, −0.77 (10.7 mmol L^−1^) for Kelex 100 concentration and 0.817 (pH = 7.4) for the donor pH. Nonetheless, in the following experiments the pH of the acceptor phase was maintained at a value of 2.0 with the aim of avoiding high depletion of the donor phase (see below), and carrier concentration was set at 12 mmol L^−1^, which is close to the recommended value, and represents a good compromise to sample at different pH values of the donor phase.

### 2.3. Hydrodynamic Conditions

The influence of different hydrodynamic regimens on E was evaluated using three different agitation forms: orbital shaking, stirring, and wrist-action shaking. The obtained values of 544, 657, and 447, respectively, showed a slightly lower value when using the wrist-action shaking; however the strong shear force produced by this operation mode tend to compete with the capillary forces that maintain the organic phase within the pores of the HFSLM, making it unstable. For this reason further experiments were performed using orbital shaking. Additionally, this mode more likely reproduces the environmental situation where the module is expected to operate.

### 2.4. Nickel Concentration in the Donor Phase

The results of the variation of metal ion content in the donor solution are shown in Table 2. As observed, the decrease in metal content increases E. This result compares to those observed in similar systems using Ni(II) and other metal ions [24,28] and may be related to kinetics effects governing metal ion transport.

**Table 2:** Influence of nickel concentration on its enrichment factor. Donor phase: 0.01 mol L^−1^ MES, pH 6.6; acceptor phase: 0.01 mol L^−1^ HCOOH, pH = 2; membrane phase: 0.012 mol L^−1^ Kelex 100; 300 min of sampling time (n = 3).

**Ni(II) mol L^−1^**	**E**
8.0 × 10^−5^	120
1.2 × 10^−5^	400
5.5 × 10^−6^	1300
2.5 × 10^−6^	1580
6.6 × 10^−7^	3530
2.5 × 10^−7^	4240

### 2.5. Presence of Inorganic Ions in the Donor Phase

**Figure 2:**
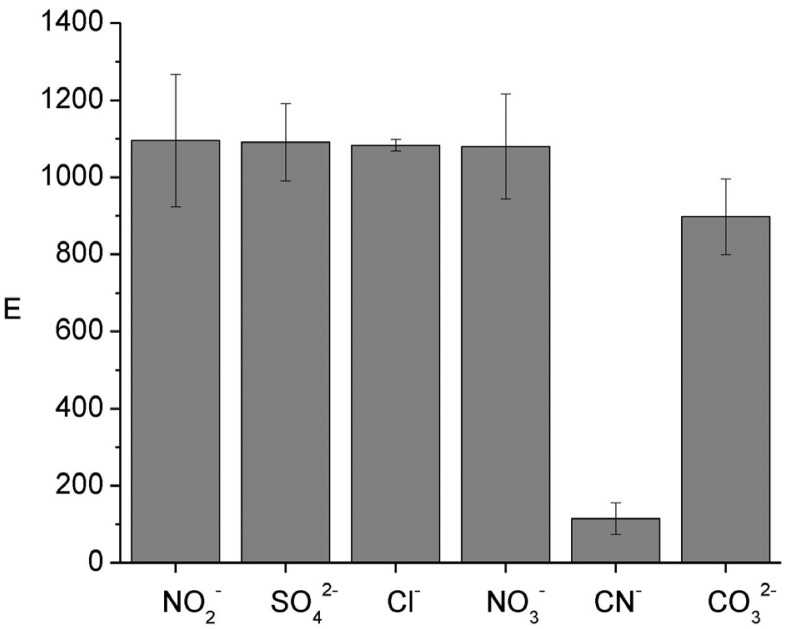
Influence of 50 mg L^−1^ of NO_2_^−^, SO_4_^2−^ Cl^−^, NO_3_^−^, CO_3_^2−^ and 5 mg L^−1^ of CN^−^ on the enrichment factor. Donor phase: 6.2 × 10^−6^ mol L^−1^ Ni(II), 0.01 mol L^−1^ MES, pH 6.6; acceptor phase: 0.01 mol L^−1^ HCOOH, pH = 2; membrane phase: 0.012 mol L^−1^ Kelex 100; 300 min of sampling time (n = 3).

To evaluate how E is affected by inorganic ions in the donor phase experiments in the presence of 50 mg L^−1^ of NO_2_^−^, SO_4_^2−^, Cl^−^, NO_3_^−^, CO_3_^2−^ and 5 mg L^−1^ of CN^−^ were carried out. From Figure 2 it is observed that E remains constant for all ions, except CN^−^, which decreases E considerably. Taking into account the Eigen mechanism, in which formation of an outer-sphere complex is followed by the rate-limiting loss of inner-sphere coordination water of the metal [29,30], and the low value of the water exchange constant of nickel (k = 3 × 10^4^ s^−1^, [31]), a low complexation kinetics of the metal ion is expected. Such behavior is likely to influence its speciation in natural systems [7]. However, once equilibrium is reached, the difference in the complexation constants values is responsible for the observed behavior (SO_4_^2−^, log β_1_ = 2.32; Cl^−^, log β_1_ = -0.21; NO_3_^−^, log β_1_ = −0.30, log β_2_ = −0.60; CN^−^, log β_4_ = 30.22 [32]). Clearly, cyanide forms the strongest complex of the studied anions reducing the E value due to the masking effect toward nickel, *i.e.*, it behaves as an inert complex in the system. Additional experiments also showed that the increase in cyanide content further reduces the enrichment factor. The formation of the nickel-cyanide complex has been reported to markedly reduce the toxicity of both cyanide and nickel at high concentrations in alkaline pH although at lower concentrations and acidic pH, nickel-cyanide solutions increased in toxicity by more than 1000 times, owing to dissociation of the metallocyanide complex to form hydrogen cyanide [33].

### 2.6. Presence of Dissolved Organic Matter (DOM)

**Figure 3:**
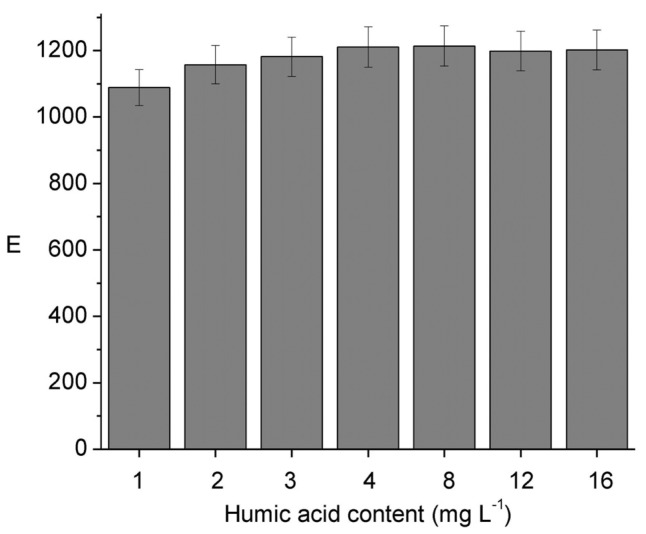
Influence of DOM in the form of humic acids at variable concentrations on the enrichment factor. Donor phase: 6.2 × 10^−6^ mol L^−1^ Ni(II), 0.01 mol L^−1^ MES, pH 6.6; acceptor phase: 0.01 mol L^−1^ HCOOH, pH = 2; membrane phase: 0.012 mol L^−1^ Kelex 100; 300 min of sampling time.

The influence of DOM on E was evaluated using humic acid (HA) as a model compound. Results from Figure 3 show no variation of E with the increase in HA concentration up to a value of 16 mg L^−1^, which is in the upper limit to be found in drinking water sources [34,35,36,37]. Considering the lowest bonding strength to organic matter reported for Ni in comparison to other metal ions like Pb, Zn, Cu, and Cd [38] this result makes sense. Furthermore, although a decrease in the dissociation rate of Ni(II)–HA complexes is expected as the [Ni(II)]/[HA] mole ratio decreases according to Guthrie *et al.* [6], the dissociation is highly dependent on pH, due to the different acid groups in HA with numerous pKa values. The absence of variation of E on the humic acid concentration also agrees with the reported evidence that increasing dissolved organic content (from approximately 1 to 30 mg L^−1^) had no effect on the 48 h Ni LC_50_ for H. Azteca [37].

### 2.7. Influence of Temperature

Temperature was varied in the 15–35 °C (288.15–308.15 K) range and its effect on E evaluated. The linear equation describing the found dependence is:

(3) $$\text{E}(\text{K})=-8617.3+30.5\text{T}({\text{r}}^{2}=0.992)$$

**Figure 4:**
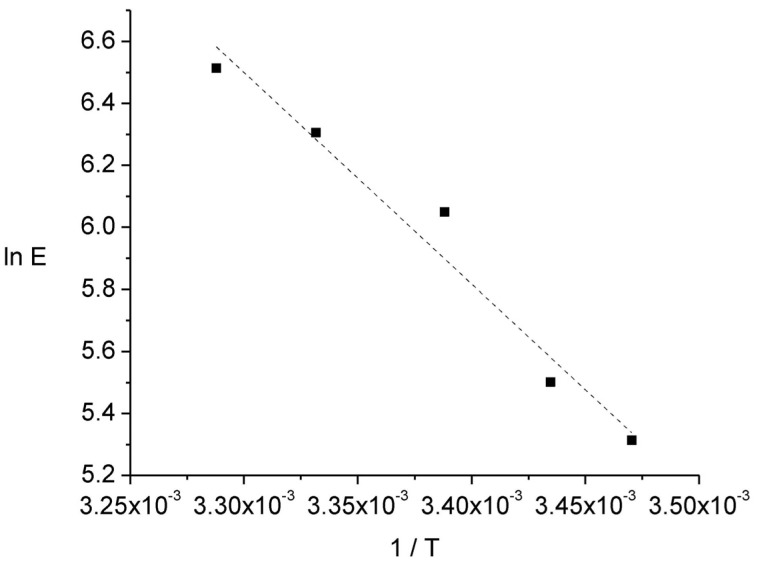
Arrhenius-type plot from which the activation energy of the permeation process, E_a_, was evaluated. Donor phase: 6.7 × 10^−6^ mol L^−1^ Ni(II), 0.01 mol L^−1^ MES, pH 6.6; acceptor phase: 0.01 mol L^−1^ HCOOH, pH = 2; membrane phase: 0.012 mol L^−1^ Kelex 100; 300 min of sampling time.

Equation (3) indicates an increase in E with the increase in temperature. This behavior may be explained considering an increase in the mobility of the migrating species or an increase in the rate of the chemical reactions responsible for permeation as temperature increases. To bring light upon the type of transport regime, experimental results were fitted to an Arrhenius-type equation [28,39]:

(4) $${\text{J}}_{\text{acceptor}}={\text{J}}_{0}\exp\left(-\frac{{\text{E}}_{\text{a}}}{\text{RT}}\right)=\text{kE}$$

where J_acceptor_ is the nickel flux entering the acceptor solution, J_0_ a pre-exponential factor, E_a_ the apparent activation energy, R the universal gas constant (8.314 J K^−1^ mol^−1^), and T the temperature in Kelvin. As E is directly proportional to J_acceptor_ by the k constant [28], the E_a_ value can be computed at once from the ln E *vs.* 1/T plot. From Figure 4 a value of 56.7 kJ mol^−1^ was calculated. As in general the activation energy of a physical process is less than 20 kJ mol^−1^ while that of a chemical process exceeds 40 kJ mol^−1^ [40] the obtained value suggests that the permeation rate might be controlled by a kinetic regime, *i.e.*, one or more of the chemical steps of the reaction mechanism (metal dehydration, complexation/decomplexation with the carrier) is rate-determining.

### 2.8. Influence of the Acceptor Solution pH and Concentration

**Figure 5:**
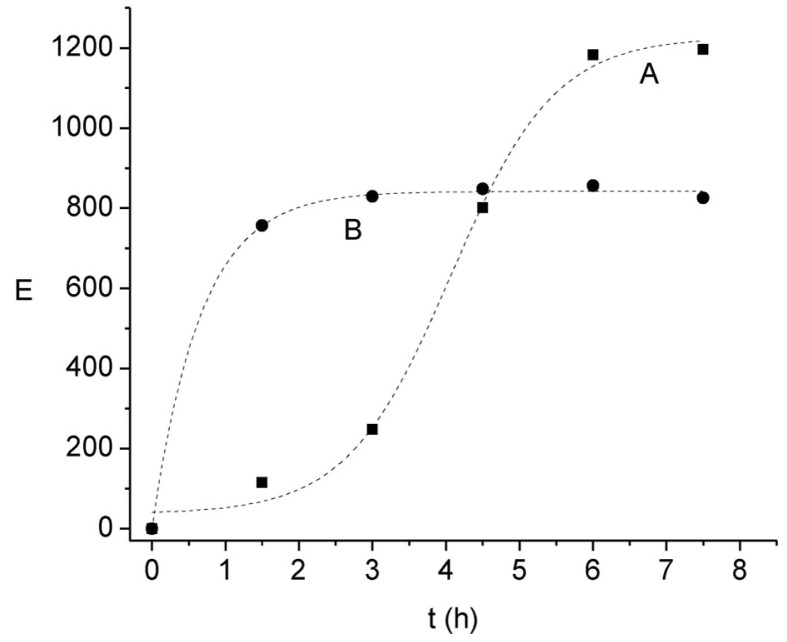
Influence of the composition of the acceptor solution on the enrichment factor. Donor phase: 6.6 × 10^−6^ mol L^−1^ Ni(II), 0.01 mol L^−1^ MES, pH 6.6; membrane phase: 0.012 mol L^−1^ Kelex 100; acceptor phase: 0.01 mol L^−1^ HCOOH, pH = 1 (**A**), and 0.1 mol L^−1^ HCOOH, pH = 2 (**B**).

Although previous results have already shown that the pH of the acceptor solution exerts a significant influence on E, due to the stagnant nature of this phase it is likely that the principal transport resistance may be related to the back-extraction rate in the kinetic regime pointed out above. This statement is also supported considering both the stoichiometry of interchange between H^+^ and Ni(II) and the proton reservoir amount, which would lead to much higher E values than those observed, indicating a limitation in the proton exchange mechanism in the acceptor phase. To further confirm this assumption the kinetic profiles of E at two acceptor solution pH values and formic acid concentrations are compared in Figure 5. It is clearly observed that both the time-dependant profile of E and the maximum E value differ in each condition. As expected, an increase in the pH gradient between the acceptor and donor phases increases the enrichment; on the other hand, the increase in the complexing agent concentration in the acceptor phase favors a faster initial migration of the cation.

### 2.9. Sample Depletion

**Figure 6:**
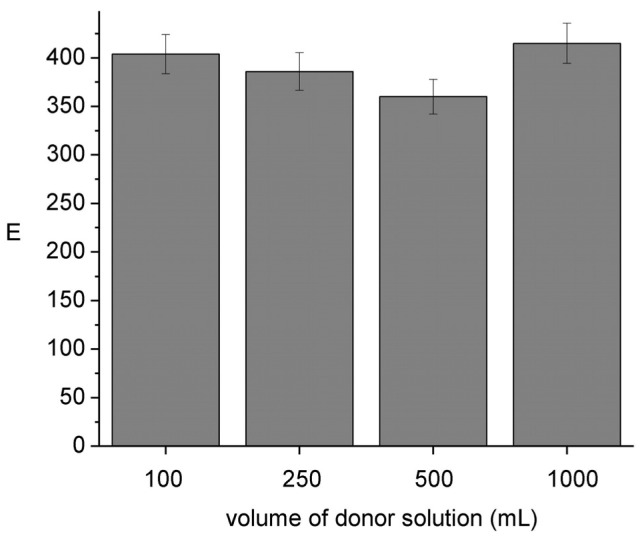
Influence of the sampling volume on the enrichment factor. Donor phase: 1.2 × 10^−5^ mol L^−1^ Ni(II), 0.01 mol L^−1^ MES, pH 6.6; acceptor phase: 0.01 mol L^−1^ HCOOH, pH = 2; membrane phase: 0.012 mol L^−1^ Kelex 100; 300 min of sampling time.

To be able to use the HFSLM device without disturbing the chemical composition of the sample it is important to assure its negligible depletion [4]. Results shown in Figure 6 indicate that E remains constant independent of the sample volume; sample depletion percents ranging from 12 (100 cm^3^) to 1.2 (1000 cm^3^) were evaluated from the analysis of the donor and acceptor pH compositions after sampling. These results ensure negligible depletion conditions (<5%) when the module is operated in lakes, rivers, *etc*.

## 3. Experimental Section

### 3.1. Reagents and Apparatus

Nickel(II) donor solutions were prepared by dilution from an atomic spectroscopy standard (Fisher Scientific, Waltham, MA, USA); sodium nitrite (Merck, Darmstadt, Germany), sodium sulfate (Mallinckrodt Baker, Lopatcong Township, NJ, USA), sodium chloride (Jalmek Científica, San Nicolás de los Garza, Monterrey, Mexico), potassium cyanide (Merck), potassium nitrate (Merck), calcium carbonate (Merck) and humic acid (ash∼20, Fluka (Sigma Aldrich Corporation, St. Louis, MO, USA)) were used when the influences of inorganic anions and organic matter were studied. Formic acid (Mallinckrodt Baker) was employed as acceptor solution. 7-(4-Ethyl-1-ethylocty)-8-hydroxyquinoline (Kelex 100, Sherex Chemical Co. Inc, Dublin, OH, USA) was employed as carrier, *i.e.*, organic ligand for Ni compexation in the membrane phase, dissolved in 1-phenylhexane (Sigma Aldrich Corporation) as organic solvent.

50/280 Accurel PP polypropylene hollow fiber tubing (50 μm wall thickness, 280 μm inner diameter and 0.1 μm pore size) was purchased from Membrana GmbH (Wuppertal, Germany) and manually cut into 15 cm-long pieces to make the membrane devices. BD Micro-Fine Syringes were employed to fill and withdraw the acceptor solution from the hollow fiber. Nitric acid (Mallinckrodt Baker), sodium hydroxide (Sigma Aldrich Corporation), and 4-morpholine ethanesulfonic acid (MES, Sigma Aldrich Corporation, pH 5.5–6.7) were used to adjust the pH of the donor solutions. Deionized water was obtained from a Milli-Q Gradient water system (Millipore, Billerica, MA, USA) and was used to prepare all solutions and to rinse all glassware after cleaning it with nitric acid for several days.

A 3100 Perkin Elmer Atomic Absorption Spectrometer (Waltham, MA, USA) allowed metal quantification in the aqueous solutions. A 211 Microprocessor pH Meter was used to adjust the pH of the donor and acceptor solutions using a 3 in 1 combo w/RJ electrode. An IKA KS 260 control S1 orbital Shaker, a Burrell 75 wrist-action shaker and a Nuova II Thermolyne stirring plate were used. Statgraphics Plus 4.0 (Statistical Graphics, Rockville, MD, USA) was employed for data analysis.

### 3.2. Membrane Preparation and Experimental Set-Up

SLMs were impregnated using the following sequence of steps: (i) filling the lumen of the hollow fiber with the acceptor solution, (ii) joining the ends of the module with aluminum foil and inserting into a small glass piece which acts as an anchor, (iii) dipping the hollow fiber into the solvent to form the liquid membrane, and (iv) the membrane was retracted from the organic phase and shaken manually to remove its excess. The pH of the formic acid (Mallinckrodt Baker) solutions employed as acceptor phases were fixed using nitric acid or sodium hydroxide as required, while that of the donor solution was buffered using 0.01 mol L^−1^ MES. Once the HFPLM devices were ready, they were immersed into 100 mL of donor solution (prepared 24 h before) with fixed composition using a 500 mL recipient with rectangular shape and shaken for stated times, except in those experiments in which the volume of the source solution was varied as indicated in the text. Initial nickel concentrations were selected so that the analysis procedure of the solutions could be performed using F-AAS.

### 3.3. Analysis Procedure

After performing the sampling experiments, the hollow fiber module was removed, the acceptor solution flushed out with the syringe and introduced into a small test tube, weighted, diluted by weight (about 1–2 mL), and collected for further analysis by FAAS.

## 4. Conclusions

Nickel(II) preconcentration using a HFSLM device containing Kelex 100 as carrier and formic acid/nitric acid as acceptor phase was satisfactorily achieved. Preconcentration factors, E, as high as 4240, were observed depending on the experimental parameters (acceptor pH, carrier concentration, pH of the donor phase, and nickel concentration). E was independent of the presence of 50 mg L^−1^ of NO_2_^−^, SO_4_^2−^, Cl^−^, NO_3_^−^, and CO_3_^2−^; although 5 mg L^−1^ of CN^−^ diminished considerably its value. Furthermore, in the presence of up to 16 mg L^−1^ of humic acids a constant value of E was obtained. It was observed that the increase in temperature increases E according to the equation E(K) = −8617.3 + 30.5T, due to the enhancement in the rate of the chemical reactions responsible for permeation. The transport process was characterized to be under kinetic control with activation energy of 56.7 kJ mol^−1^. The stagnant nature of the acceptor phase suggests that the back-extraction kinetics play a major role, as it was confirmed in experiments in which the composition of this phase was varied. Sample depletion ranged from 12 to 1.2% depending on the volume of the donor phase (100 to 1000 cm^3^, respectively); accordingly, negligible depletion is expected when sampling natural samples, e.g., lakes and rivers, making the HFSLM device useful for nickel speciation analysis. Further work aimed to evaluate the analytical performance characteristics of the method and its validation is needed before applying the procedure to natural samples.
